# Genetic characterisation of a subset of *Campylobacter jejuni* isolates from clinical and poultry sources in Ireland

**DOI:** 10.1371/journal.pone.0246843

**Published:** 2021-03-09

**Authors:** Brendha Truccollo, Paul Whyte, Catherine Burgess, Declan Bolton

**Affiliations:** 1 Food Safety Department, Teagasc Food Research Centre, Dublin, Republic of Ireland; 2 School of Veterinary Medicine, University College Dublin, Dublin, Republic of Ireland; Cornell University, UNITED STATES

## Abstract

*Campylobacter spp*. is a significant and prevalent public health hazard globally. *Campylobacter jejuni* is the most frequently recovered species from human cases and poultry are considered the most important reservoir for its transmission to humans. In this study, 30 *Campylobacter jejuni* isolates were selected from clinical (n = 15) and broiler (n = 15) sources from a larger cohort, based on source, virulence, and antimicrobial resistance profiles. The objective of this study was to further characterise the genomes of these isolates including MLST types, population structure, pan-genome, as well as virulence and antimicrobial resistance determinants. A total of 18 sequence types and 12 clonal complexes were identified. The most common clonal complex was ST-45, which was found in both clinical and broiler samples. We characterised the biological functions that were associated with the core and accessory genomes of the isolates in this study. No significant difference in the prevalence of virulence or antimicrobial resistance determinants was observed between clinical and broiler isolates, although genes associated with severe illness such as *neuABC*, *wlaN* and *cstIII* were only detected in clinical isolates. The ubiquity of virulence factors associated with motility, invasion and cytolethal distending toxin (CDT) synthesis in both clinical and broiler *C*. *jejuni* genomes and genetic similarities between groups of broiler and clinical *C*. *jejuni* reaffirm that *C*. *jejuni* from poultry remains a significant threat to public health.

## 1. Introduction

*Campylobacter* is the most prevalent bacterial foodborne zoonosis globally, with over 200,000 cases reported annually in the European Union [1]. Most infections are generally self-limiting and consist of diarrhoea (which may be watery or haemorrhagic), myalgia, abdominal cramps, while fever, nausea and vomiting may also be present [2, 3]. In most cases, symptoms first appear within 48h of ingestion and subside after 7–10 days without medical intervention [4]. More serious complications can arise in a subset of cases, which include Guillain Barré Syndrome, Miller Fisher Syndrome, reactive arthritis, irritable bowel syndrome, inflammatory bowel disease, and bacteraemia [3].

The natural environment of *Campylobacter* is the gastrointestinal tract of birds and mammals, and it is primarily transmitted to humans through the handling and consumption of contaminated broiler meat [5]. *Campylobacter* is generally introduced into the broiler production cycle two to three weeks after hatching, and rapidly spreads within the flock [6, 7]. This contamination persists until slaughter, and it is frequently found on processed carcases and raw chicken, posing a risk to public health [8].

*Campylobacter* persistence and transmission in the broiler environment and to human hosts depends on the ability of these pathogens to tolerate oxidative, osmotic, desiccation and thermal stress, to compete with endogenous microflora and to evade host immune responses. Stresses encountered by campylobacters in the environment have resulted in the development of a variety of survival and adaptation mechanisms, which can arise due to genetic mutations or the acquisition of genetic material from the environment [9]. Passage through the gastrointestinal tract of chicks has been previously associated with increased adherence, while passage through mice has been associated with increased invasiveness [10, 11]. Additionally, passage through human and murine hosts has also lead to the acquisition of genetic mutations, while passage through chicks may result in phase variation that is strongly associated with colonisation and disease [10–13]. *Campylobacter* is highly genetically diverse due to frequent recombination, which can confer different virulence and survival abilities [9]. Additionally, horizontal gene transfer can lead to the acquisition of virulence and antimicrobial resistance determinants [14–17], and in some cases mobile genetic elements may become chromosomally integrated [18].

In this study our objective was to examine a small subset of *C*. *jejuni* strains from broilers and humans infection in Ireland by characterising the genotypes, gene content, and virulence and antimicrobial resistance determinants in their respective genomes.

## 2. Materials and methods

### 2.1 Bacterial strain cultivation

Thirty *Campylobacter jejuni* isolates from clinical stool samples (n = 15) and broiler caecal samples (n = 15) were selected based on their virulence profiles previously determined by conventional PCR-based amplification of the *dnaJ*, *racR*, *cdtA*, *cdtB*, *cdtC*, *ciaB*, *pldA*, *flaA*, *flaB* and *tet(O)* genes, antimicrobial resistance profiles, and source. They are listed in **Table 1**. Each isolate represents a distinct virulence profile which was found within a larger cohort of clinical and broiler isolates, respectively. The isolates were resuscitated from defibrinated horse blood stored at -80°C on modified charcoal cefoperazone deoxycholate agar (mCCDA, Oxoid, UK) containing a *Campylobacter* selective supplement (SR0155E, Oxoid, UK), followed by microaerobic incubation at 42°C for 48h. Pure single colonies were subcultured onto Mueller Hinton agar supplemented with 5% defibrinated horse blood (MHAb) and incubated microaerobically at 42°C for 48h.

**Table 1:** Sources of the clinical and broiler *C*. *jejuni* isolates selected for this study.

Isolate ID	Host	Source	Year
CJC01	Human	Non-outbreak patient	2017
CJC02	Human	Non-outbreak patient	2016
CJC03	Human	Non-outbreak patient	2016
CJC04	Human	Non-outbreak patient	2016
CJC05	Human	Non-outbreak patient	2017
CJC06	Human	Non-outbreak patient	2016
CJC07	Human	Non-outbreak patient	2016
CJC08	Human	Non-outbreak patient	2016
CJC09	Human	Non-outbreak patient	2017
CJC10	Human	Non-outbreak patient	2017
CJC11	Human	Non-outbreak patient	2016
CJC12	Human	Non-outbreak patient	2017
CJC13	Human	Non-outbreak patient	2017
CJC14	Human	Non-outbreak patient	2016
CJC15	Human	Non-outbreak patient	2016
CJB16	Broiler	Farm	2016
CJB17	Broiler	Farm	2013
CJB18	Broiler	Farm	2013
CJB19	Broiler	Farm	2008
CJB20	Broiler	Farm	2013
CJB21	Broiler	Farm	2008
CJB22	Broiler	Farm	2008
CJB23	Broiler	Farm	2008
CJB24	Broiler	Farm	2008
CJB25	Broiler	Farm	2008
CJB26	Broiler	Farm	2008
CJB27	Broiler	Farm	2008
CJB28	Broiler	Farm	2014
CJB29	Broiler	Farm	2008
CJB30	Broiler	Farm	2008

### 2.2 DNA extraction

A 1μl loopful of each MHAb culture was transferred to 500μl of phosphate-buffered saline, which was briefly vortexed and centrifuged at 5,000 x g for 10min. Supernatant was removed and the pellet was homogenised in 180μl of Buffer ATL (Qiagen, UK) with 20μl of Proteinase K *via* gentle pipetting. Samples were incubated at 56°C until total cell lysis was achieved. Subsequent washing steps and DNA elution was carried out following the manufacturer’s instructions (DNeasy Blood & Tissue Kit, Qiagen, UK). DNA was further purified and concentrated *via* precipitation with the addition of 2.5 volumes of ice-cold 100% ethanol and 0.1 volumes of sodium acetate, pH 8.5. Each sample was kept at -20°C overnight and subsequently centrifuged at 3,400 x g for 15min. The supernatant was discarded and replaced with 500μl of 70% ethanol. Pellets were vortexed, and each sample was centrifuged for 8min at 3,400x g. The supernatant was discarded, and the DNA pellets were allowed to fully dry prior to resuspension in 50μl of nuclease-free water. The DNA concentration of each sample was standardised to a concentration of between 10 and 100ng/ul using a Nanodrop device. Samples with 260/280 and 260/230 ratios greater than 1.8 were submitted for whole genome sequencing, and were stored at -20°C until needed.

### 2.3 Whole genome sequencing

Sequencing was carried out by MicrobesNG (Birmingham, UK). As per their protocol, following quantification using a Quant-IT dsDNA HS assay in an Eppendorf AF2200 plate reader, genomic DNA libraries were prepared using Nextera XT Library Prep Kit (Illumina, San Diego, USA) in accordance with the manufacturer’s protocol with the following modifications: two nanograms of DNA instead of one were used as input, and the PCR elongation time was increased from 30 seconds to 1 minute. DNA quantification and library preparation were carried out on a Hamilton Microlab STAR automated liquid handling system. Pooled libraries were quantified using the Kapa Biosystems Library Quantification Kit for Illumina on a Roche LightCycler 96 qPCR machine. Libraries were sequenced on the Illumina HiSeq using a 250bp paired-end protocol.

### 2.4 Bioinformatic analysis

Reads were adapter trimmed using Trimmomatic 0.30 with a sliding window quality cut-off of Q15 [19]. They were *de novo* assembled on SPAdes v.3.14 with the—careful command [20]. The quality of the assemblies was assessed on QUAST v5.0.2 using *C*. *jejuni* subsp. *jejuni* NCTC 11168 as a reference [21, 22]. Kraken v.2.0 was used for taxonomic classification [23]. Annotation was carried out in Prokka v.1.14 with -usegenus and -genus *Campylobacter* parameters [24]. Multilocus sequence typing (MLST) was carried out on PubMLST on the BIGSdb database [25].

Annotated genomes were separated into three groups for pan-genomic analysis on Roary [26]: one group comprising all 30 genomes in the dataset; clinical, comprising 15 *C*. *jejuni* genomes from clinical human infections; and broiler, comprising the remaining 15 *C*. *jejuni* genomes isolated from broilers. The purpose of this was for analysing the overall pangenome of these isolates and to compare differences in the pangenome of the clinical and broiler *C*. *jejuni* genomes in this study. Roary was run with standard parameters and without paralog splitting. The query_pan_genome command was used to produce files containing information on the core and accessory genes of each group. To isolate the genes that were uniquely present in clinical *C*. *jejuni* or broiler *C*. *jejuni* genomes, query_pan_genome -a difference was used. Sequences for all core, accessory and unique genes within each group were extracted from the pan_genome_reference.fa output. The sequences were concatenated and submitted to eggNOG Mapper v.2 for functional annotation using default parameters and with a taxonomic scope limited to Epsilonproteobacteria [27]. The outputs of this analysis can be found in **S2 Appendix**. The pangenome results were visualised on Phandango using the gene_presence_absence.csv and Newick tree outputs from Roary [28], while accumulation curves were constructed on RStudio v.1.1.463 (RStudio Inc., Massachussets, US) using the create_pan_genome_plots.R script [26].

Whole genome differences were visualised by aligning each assembled genome to NCTC 11168 on the Blast Ring Image Generator (BRIG), with an upper identity of 70% and a lower identity of 50% and default parameters [29].

The core_genome_alignment.aln output from Roary was used to generate a maximum likelihood tree on RaxML v.8.2.12 with 500 bootstraps with standard parameters and -m GTRGAMMA -p 12435 -f -a and -x 12345 commands [30]. The resulting tree was visualised on iTOL v.5 [31]. To create a distance matrix, the core genome alignment was read on RStudio v.1.1.463 (RStudio Inc., Massachussets, US) using *adagenet*, and genetic distances were computed using *ape* equipped with Tamura and Nei 1993’s model [32–34].

ABRicate (https://github.com/tseemann/abricate) equipped with VFDB and Victors was used to screen for genes associated with virulence factors including motility [35, 36], adhesion, invasion, colonisation, lipooligosaccharide (LOS) synthesis, capsule synthesis and thermotolerance (Chen et al., 2016; Sayers et al., 2019). Antimicrobial resistance was screened using ABRicate equipped with ARG-ANNOT, CARD, Resfinder and NCBI AMRFinder Plus (Feldgarden et al., 2019; Gupta et al., 2014; Jia et al., 2017; Zankari et al., 2012). Discrepancies in the assignment of antimicrobial resistance genes between databases were addressed by selecting the gene with the highest coverage and identity. For both AMR and virulence datasets, genes with less than 80% coverage or identity were discarded. The raw outputs for these analyses are provided in **S3 Appendix**.

ISFinder was used to investigate the presence of insertion sequences in the genomes of the isolates in this study. Results were filtered by e-value (<0.001), bit score (>40) and identity (90%) [37].

Assemblies were filtered to remove contigs <200bp using BBMap v.38.22 [38] prior to submitting each genome to Bioproject PRJNA665357.

### 2.5 Statistical analysis

Significant differences in the pangenomes calculated on RStudio v.1.1.463 (RStudio Inc., Massachussets, US) were determined with a chi-square test and a significance threshold of p < 0.05. Differences between the core genomes, accessory genomes and unique genomes of all, clinical and broiler groups were calculated, as well as differences between the core, accessory, and unique genomes within each group.

The chi-square test was also used to calculate differences in the prevalence of each virulence and antimicrobial resistance determinant between clinical and broiler genomes.

## 3. Results

### 3.1 General information

A total of 30 genomes were sequenced (15 human and 15 broiler), producing on average 27 contigs per genome, ranging from 11 to 110, with a mean read coverage of 107.2-fold that ranged from 50.2 to 620.6-fold. The average genome length of all isolates was 1.7 Mbp, ranging from 1.59–1.84 Mbp, and the mean GC content was 30.35%, ranging from 30.03–30.53%.

### 3.2 Population structure and MLST

**Fig 1:**
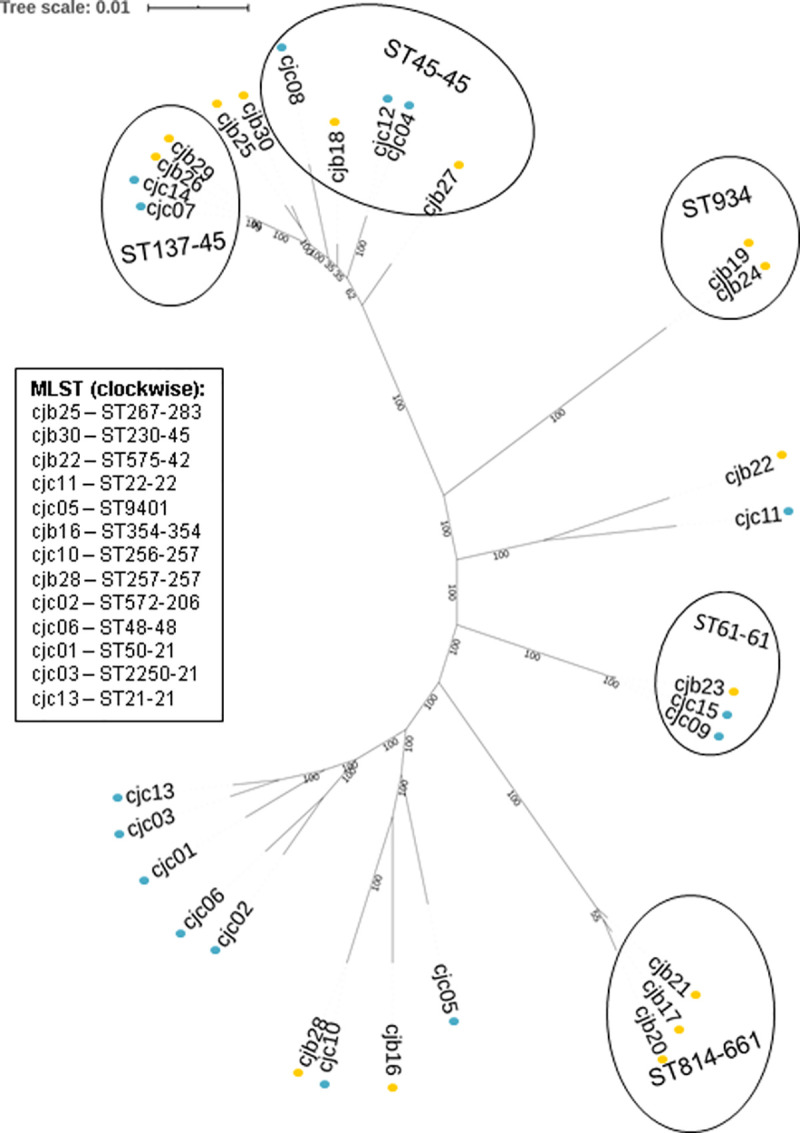
Maximum likelihood tree of *C*. *jejuni* isolates from clinical and broiler sources with bootstrap values depicted at the nodes Isolates CJC01—CJC15 were recovered from clinical human infection (blue dots), and CJB16—CJB30 were recovered from broilers (orange dots).

In total 12 different clonal complexes and 18 different sequence types (ST) were detected in this dataset (**Fig 1**). The most common MLST types were ST137-45 (n = 4) and ST45-45 (n = 5), and the most common clonal complex was ST-45 (n = 9). The most common MLST type among clinical genomes was ST45-CC45 (ST45-45; n = 3), while ST814-661 was the most common MLST type among broiler genomes (n = 3) which is likely due to the presence of three genetically similar strains from the same source in this study that originated from the same flock. The second most common MLST types in poultry were ST137-45 and ST934. The most common clonal complex in both clinical and broiler genomes was ST-45.

The maximum likelihood tree (**Fig 1**) revealed a cluster of genetically similar genomes from a mixture of clinical and broiler sources that comprised isolates with MLST types ST137-45, ST45-45, ST230-45 and ST267-283. The remaining genomes in this study were generally more distantly related, unless they shared an MLST type or clonal complex. Overall, most broiler isolates showed some similarity to the clinical isolates, with the exception of ST814-661 (n = 3) and ST934 (n = 2) clusters. Furthermore, five clinical isolates (CJC13, CJC03, CJC01, CJC06 and CJC02) clustered further away from the broiler isolates in this study. The distance matrices (**S1 Appendix**) reflect the observations from the maximum likelihood tree.

In this study, 46.6% (n = 7) of the clinical isolates shared an MLST type and clonal complex with broiler isolates, and also clustered closely together with broiler isolates. Similarly, 33.3% (n = 5) of broiler isolates shared an MLST type and 46.6% (n = 7) shared a clonal complex with clinical isolates.

### 3.3 Pan-genome

**Fig 2:**
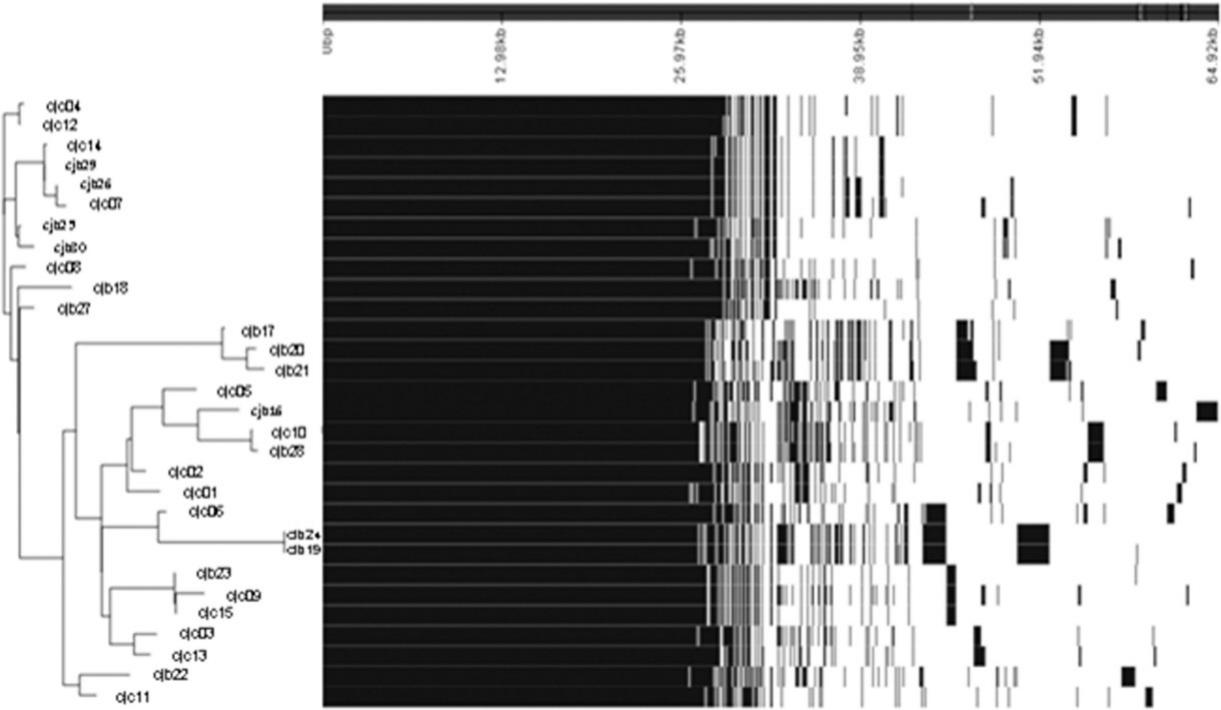
Visualisation of all genes that are present and absent throughout each genome.

A total of 3244 genes were detected in the pangenome of the 30 *C*. *jejuni* genomes in this study, of which an estimated 1327 (32.3%) constituted the core genome and 1917 (67.7%) the accessory genome (**Fig 2**). Accumulation curves (**S2 Appendix**) reveal an open pan-genome, as new genes were added to the data pool with the addition of each sample genome. As expected, the number of conserved genes decreased with the addition of sample genomes. A slight plateau was observed, which indicates that with the addition of more diverse *C*. *jejuni* genomes the core genome may not become substantially reduced.

Pan-genome estimations were also carried out by separating *C*. *jejuni* genomes by clinical and broiler sources to compare differences. The core genome of broiler isolates (n = 1346) did not substantially differ in size from the core genome of clinical isolates (n = 1368), while the accessory genome of broiler isolates (n = 1668) was considerably larger than that of clinical isolates (n = 1353). Additionally, the pan-genomes of isolates with the same MLST types were not identical (**Fig 2C**), which indicates that differences in gene content were present in closely related strains.

Functional annotation of the pangenome identified 2789 coding sequences in total, of which 87.9% were characterised into COG categories and known biological functions were assigned to 79.0% of these (**S2 Appendix**). Most coding sequences that were assigned to known biological functions were associated with cell wall biogenesis (10.6%), translation (6.7%), amino acid metabolism (6.4%), and inorganic ion metabolism (5.9%). The prevalence of functions including DNA replication, energy production, coenzyme transport and metabolism, intracellular trafficking, cell motility, nucleotide transport and metabolism, transcription, post-translational modification, defence mechanisms, lipid transport and metabolism, and signal transduction ranged from 2.1% to 5.5%. The least prevalent categories were RNA processing and modification (>0.1%), cell cycle control (0.9%), and secondary metabolite biosynthesis (1.7%).

The comparison of functional annotation of the core, accessory, and unique genomes for clinical and broiler genomes is provided in **Table 2**. Overall, a number of biological functions were associated with the core or the accessory genome and biological functions associated with the unique genome of broiler isolates were also identified.

**Table 2:** Functional annotation of the pan, core and accessory genomes and of genes unique to clinical and broiler *C*. *jejuni* isolates.

COG Category	Function	Broiler				Clinical			
		Pan (n = 2789)	Core (n = 1319)	Accessory (n = 1289)	Unique (n = 419)	Pan (n = 2410)	Core (n = 1340)	Accessory (n = 1070)	Unique (n = 223)
A	RNA processing and modification	1(0.0%)	1(0.1%)	0(0.0%)	0(0.0%)	1(0.0%)	1(0.1%)	0(0.0%)	0(0.0%)
C	Energy production and modification	145(5.2%)	117(8.9%)***^,^ ^a^	28(2.2%)	7(1.7%)	142(5.9%)	118(8.8%)***^,^ ^a^	24(2.2%)	5(2.2%)
D	Cell cycle control	25(0.9%)	17(1.3%)***^,^ ^a^	8(0.6%)	3(0.7%)	27(1.1%)	17(1.3%)*^,^ ^a^	10(0.9%)	4(1.8%)
E	Amino acid transport and metabolism	177(6.3%)	118(8.9%)***^,^ ^a^	59(4.6%)	8(1.9%)	175(7.3%)	118(8.8%)***^,^ ^a^	57(5.3%)	6(2.7%)
F	Nucleotide transport and metabolism	78(2.8%)	69(5.2%)***^,^ ^a^	9(0.7%)	2(0.5%)	79(3.3%)	69(5.1%)***^,^ ^a^	10(0.9%)	2(0.9%)
G	Carbohydrate transport and metabolism	98(3.5%)	38(2.9%)	60(4.7%)***^,^ ^a^	17(4.1%)*^,^ ^e^	89(3.7%)	38(2.8%)***^,^ ^e^	51(4.8%)***^,^ ^b^	6(2.7%)
H	Coenzyme transport and metabolism	114(4.1%)	80(6.1%)***^,^ ^a^	34(2.6%)	13(3.1%)	111(4.6%)	83(6.2%)***^,^ ^a^	28(2.6%)	8(3.6%)
I	Lipid transport and metabolism	55(2.0%)	29(2.2%)***^,^ ^b^	26(2.0%)***^,^ ^c^	11(2.6%)	49(2.0%)	30(2.2%)***^,^ ^b^	19(1.8%)***^,^ ^e^	5(2.2%)
J	Translation, ribosomal structure and biogenesis	184(6.6%)	139(10.5%)***^,^ ^a^	45(3.5%)	14(3.3%)*^,^ ^e^	174(7.2%)	140(10.4%)***^,^ ^a^	34(3.2%)	5(2.2%)
K	Transcription	72(2.6%)	41(3.1%)***^,^ ^b^	31(2.4%)***^,^ ^c^	9(2.1%)	71(2.9%)	43(3.2%)***^,^ ^b^	28(2.6%)***^,^ ^e^	5(2.2%)
L	DNA replication, recombination and repair	137(4.9%)	61(4.6%)***^,^ ^c^	76(5.9%)****^,^ ^b^	29(6.9%)*^,^ ^e^	128(5.3%)	64(4.8%)***^,^ ^d^	64(6.0%)***^,^ ^d^	15(6.7%)
M	Cell wall/membrane biogenesis	273(9.8%)	96(7.3%)	177(13.7%)***^,^ ^a^	53(12.6%)**^,^ ^e^	248(10.3%)	94(7.0%)***^,^ ^e^	154(14.4%)***^,^ ^b^	26(11.7%)
N	Cell motility	89(3.2%)	53(4.0%)***^,^ ^b^	36(2.8%)***^,^ ^c^	9(2.1%)	87(3.6%)	54(4.0%)***^,^ ^b^	33(3.1%)***^,^ ^e^	5(2.2%)
O	Post-translational modification, protein turnover, and chaperones	73(2.6%)	61(4.6%)***^,^ ^a^	12(0.9%)	1(0.2%)	75(3.1%)	61(4.6%)***^,^ ^a^	14(1.3%)	3(1.3%)
P	Inorganic ion transport	159(5.7%)	96(7.3%)***^,^ ^b^	59(4.6%)***^,^ ^c^	15(3.6%)	150(6.2%)	99(7.4%)***^,^ ^a^	44(4.1%)	9(4.0%)
Q	Secondary metabolite biosynthesis, transport and catabolism	54(1.9%)	13(1.0%)	41(3.2%)***^,^ ^a^	14(3.3%)**^,^ ^e^	43(1.8%)	13(1.0%)	30(2.8%)***^,^ ^a^	3(1.3%)
S	Function unknown	523(18.8%)	191(14.5%)	332(25.8%)***^,^ ^a^	107(25.5%)**^,^ ^e^	485(20.1%)	192(14.3%)	293(27.4%)***^,^ ^a^	70(31.4%)
T	Signal transduction	56(2.0%)	40(3.0%)***^,^ ^c^	6(0.5%)***^,^ ^b^	5(1.2%)	57(2.4%)	40(3.0%)***^,^ ^a^	7(0.7%)	2(0.9%)
U	Intracellular trafficking, secretion and vesicular transport	85(3.0%)	42(3.2%)***^,^ ^c^	54(4.2%)***^,^ ^b^	18(4.3%)	82(3.4%)	43(3.2%)***^,^ ^e^	50(4.7%)***^,^ ^b^	16(7.2%)
V	Defence mechanisms	58(2.1%)	12(0.9%)	46(3.6%)***^,^ ^a^	16(3.8%)	44(1.8%)	12(0.9%)	32(3.0%)***^,^ ^a^	7(3.1%)
N/A	Uncharacterised	337(12.1%)	94(7.1%)	215(16.7%)	94(22.4%)	242(10.0%)	97(7.2%)	145(13.6%)	32(14.3%)

Percentages represent the prevalence of genes within their respective pan, core, accessory or unique genome category.

* = p < 0.05

* = p < 0.01

*** = p < 0.001

a = chi square test was only significantly positively associated with this value

b and c = both values were significantly positively associated, where b showed a stronger association than c

d = both values were significantly positively associated equally

e = significant differences in comparisons between clinical and broiler genomes.

**Fig 3:**
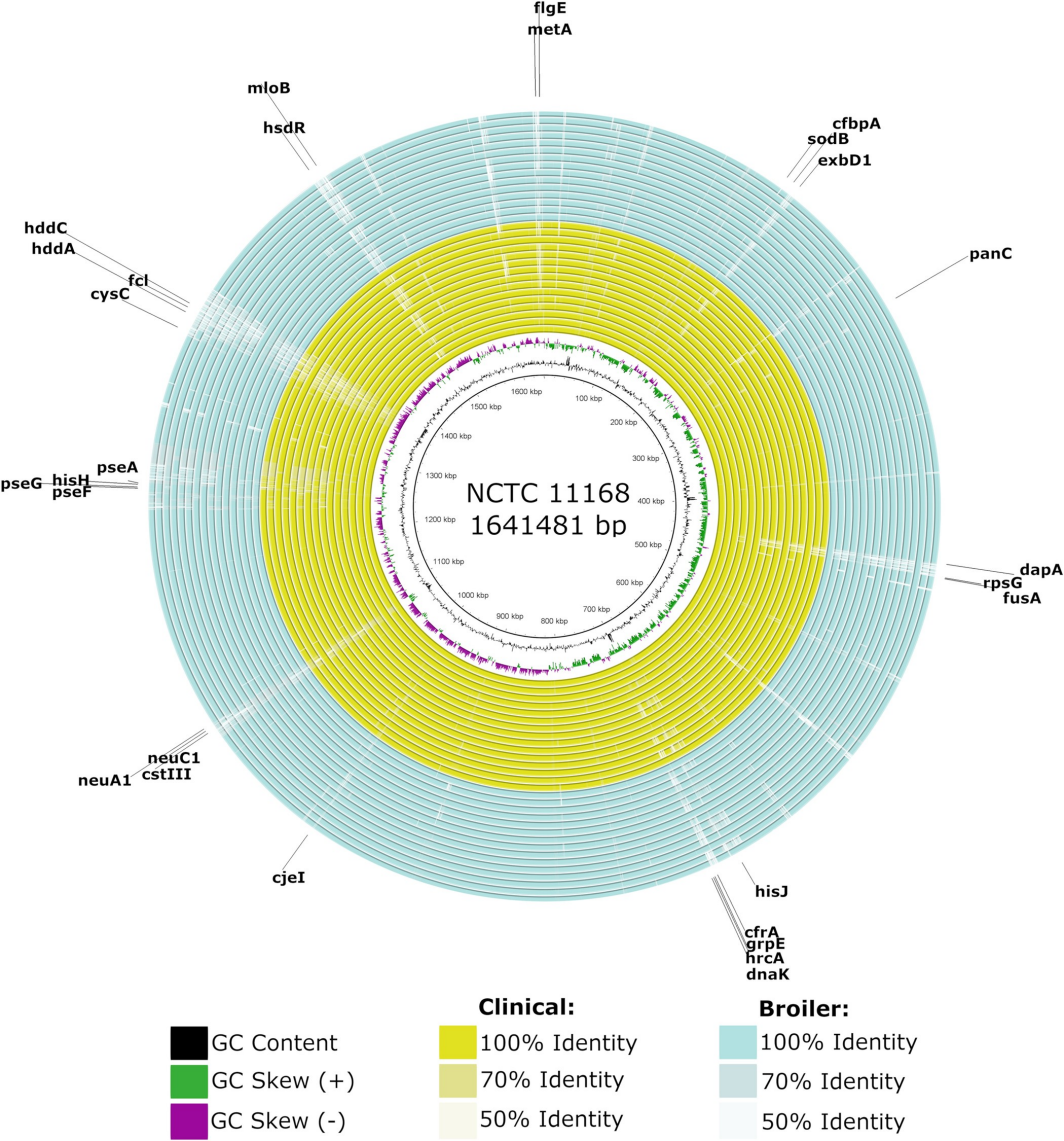
Multiple sequence alignment of human clinical and broiler *C*. *jejuni* isolates to NCTC 11168. White areas correspond to sequences with similarity values below the minimum threshold. Genomes are presented as CJC01 to CJC15 (from innermost to outermost yellow ring) and CJB16 to CJB30 (from innermost to outermost blue ring). Genes that overlap or neighbour gaps of low identity are indicated with arrows.

Alignment of each genome to NCTC 11168 (**Fig 3**) revealed gaps in some genomes in and near regions encoding genes associated with survival (amino acid metabolism—*metA*, *panC*, *dapA*, *hisJ*, *hisH*, *exbD1*; translation—*rpsG*, *fusA*; transcription—*hrcA*, *mloB*; ion metabolism—*cfbpA*, *cfrA*, *cysC*), stress response (heat shock—*grpE*, *dnaK*; acid shock—*sodB*), DNA recombination (*cjeI*, *hsdR*), motility (*flgE*, *pseA*, *pseF*, *pseG*) and cell wall biogenesis (*neuABC*, *cstII* and *III*, *fcl*, *hddA*, *hddC*), which indicates that they may have a variable distribution across these isolates.

### 3.4 Virulence

**Fig 4:**
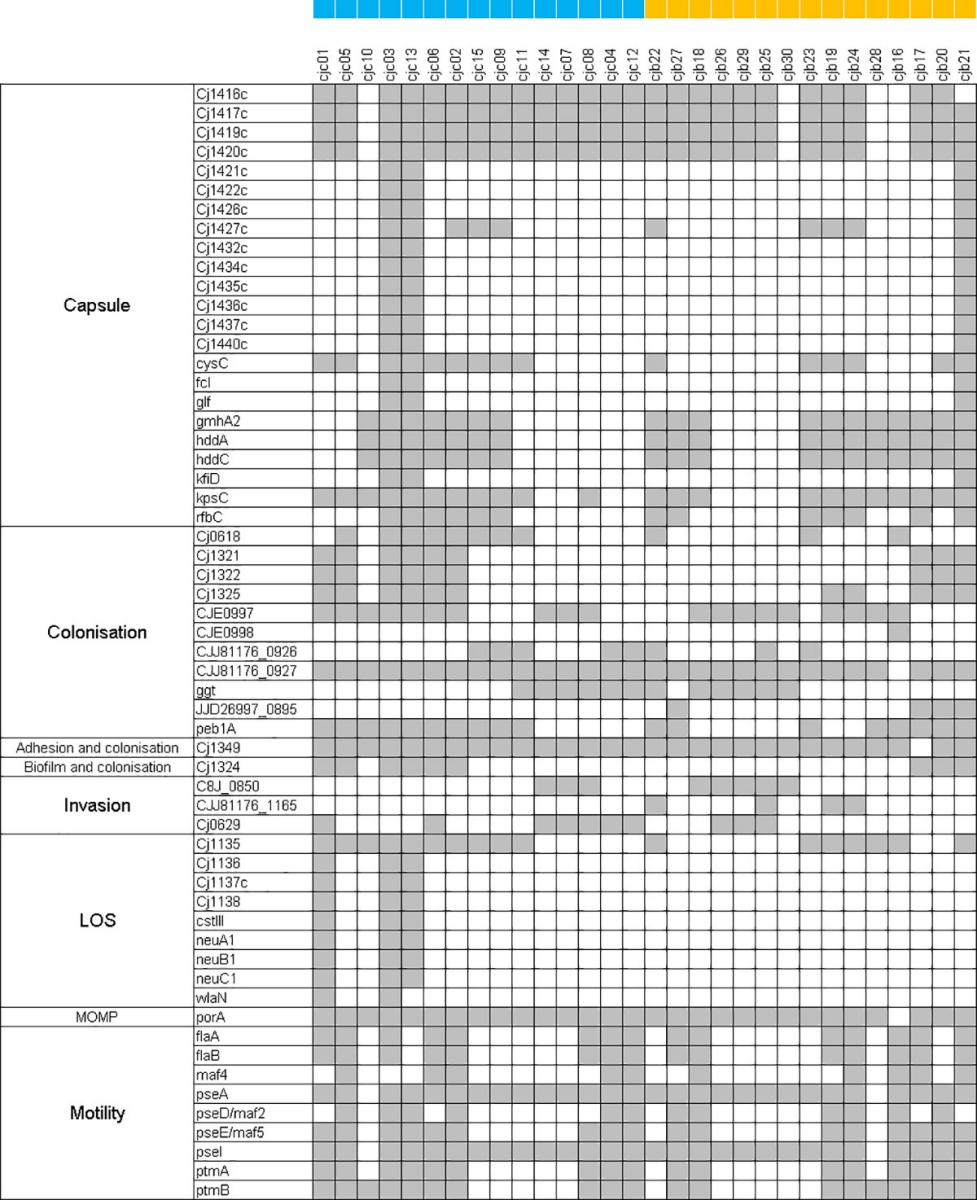
Prevalence (grey = present, white = absent) of virulence genes in broiler and human clinical *C*. *jejuni* isolates. Blue squares were placed beside clinical and orange squares were placed beside broiler isolates.

A total of 159 known virulence genes were detected in the genomes of isolates examined in this study (**S3 Appendix**). Genes with prevalence from 0 to 99% are shown in **Fig 4**. No significant difference in the prevalence of any virulence gene was detected when clinical and broiler genomes were compared (p > 0.05).

Among these genes, 63.5% were detected in all of the broiler isolate genomes, while a similar percentage (65.4%) were also present in all the clinical isolate genomes. In broiler and clinical *C*. *jejuni* genomes, the virulence associated genes that were present in all genomes, respectively, were for the most part involved in motility (49.5% and 48.1%), colonisation (13.8% and 14.4%), LOS synthesis (7.9% and 7.7%) and capsule synthesis (6.9% and 6.7%). Additionally, *cdtABC* were present in all isolates.

The remaining virulence genes with a prevalence of between 1–99% constituted 31.4% of the virulence genes found in broiler and 32.7% of the virulence genes found in clinical *C*. *jejuni* genomes. In broilers, most of these genes were involved in capsule synthesis (46%), colonisation (22%), and motility (18%), while in clinical samples most of these were associated with capsule synthesis (44.2%), LOS synthesis (17.3%), motility (17.3%), and colonisation (15.3%).

In total, 5.0% of the genes detected in this study were absent from broiler *C*. *jejuni* genomes and present in clinical genomes, all of which are involved in LOS synthesis–*Cj1136*, *Cj1137c*, *Cj1138*, *cstIII*, *neuA1*, *neuB1*, *neuC1* and *wlaN*. Notably, *cstIII*, *neuABC* and *wlaN* are involved in molecular mimicry which is associated with the development of more severe illness, including Guillain Barré Syndrome. In contrast, 1.9% of the genes detected in this study were present in broiler and absent from clinical *C*. *jejuni* genomes–*CJE0998*, *JJD26997_0895*, and *CJJ81176_1165*, which are involved in adhesion and biofilm formation.

Notably, a higher recovery rate of all virulence genes previously amplified by conventional PCR (*dnaJ*, *racR*, *cdtABC*, *ciaB*, *pldA*, *flaA* and *flaB*) was observed via WGS screening.

### 3.5 Antimicrobial resistance

**Fig 5:**
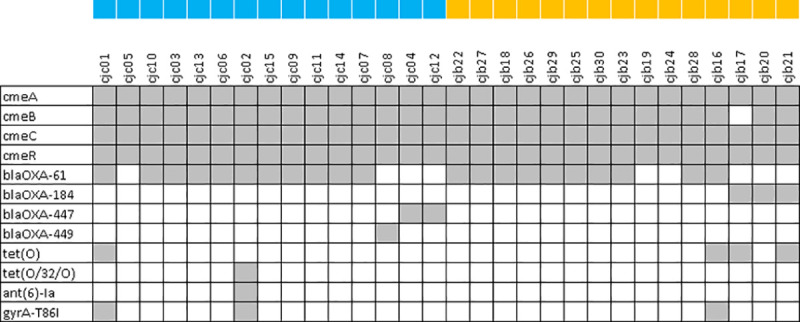
Prevalence (grey = absent, white = present) of antimicrobial resistance determinants in clinical and broiler isolates. Blue squares were placed beside clinical and orange squares were placed beside broiler isolates.

The presence of twelve antimicrobial resistance determinants was detected in the genomes of the isolates analysed in this study (**Fig 5**). No significant difference in the prevalence of antimicrobial resistance determinants was detected between clinical and broiler isolate genomes (p > 0.05).

Multidrug efflux pump genes *cmeABC* and *cmeR* were detected in all of the clinical *C*. *jejuni* genomes, while *cmeA*, *cmeC* and *cmeR* were detected in 100% of broiler *C*. *jejuni* genomes and *cmeB* was detected in all but one genome where it was identified, but below the identity threshold of 80%. β-lactam resistance genes were also prevalent and were detected in 14 out of 15 clinical *C*. *jejuni* genomes (93.3%). The most common β-lactamase in clinical *C*. *jejuni* genomes was *bla*_*OXA-61*_ (n = 11), followed by *bla*_*OXA-447*_ (n = 2), and *bla*_*OXA-449*_ (n = 1). A similar prevalence of β-lactam resistance genes was detected in the poultry *C*. *jejuni* genomes (86.7%, n = 13), wherein *bla*_*OXA-61*_ was also the most common determinant (n = 10), followed by *bla*_*OXA-184*_ (n = 3).

Less commonly found antimicrobial resistance determinants found in clinical *C*. *jejuni* genomes included *gyrA*-T86I (n = 2), *tet(O)* and *tet(O/32/O)* (where n = 1 for each), and *ant(6)-Ia* (n = 1); which are responsible for fluoroquinolone, tetracycline, and aminoglycoside resistance, respectively. In broiler *C*. *jejuni* genomes, *tet(O)* was found in three genomes, while *gyrA*-T86I was found in one. Notably, all tetracycline and ciprofloxacin resistance determinants detected matched the previously established phenotypic resistance patterns exactly.

Multidrug resistance, which is classified as resistance to three or more different classes of antimicrobials, was detected in two clinical isolates and one broiler isolate. The clinical isolates, CJC01 and CJC02, carried determinants for β-lactam, quinolone and tetracycline resistance, and for β-lactam, quinolone, tetracycline and aminoglycoside resistance, respectively, while the broiler isolate–CJB16 carried determinants for β-lactam, fluoroquinolone and tetracycline resistance.

A number of mutations that are not currently associated with antimicrobial resistance were found in *gyrA*, 23S rRNA, *cmeR* and *rpsL* genes. In total, thirty-eight mutations were found in 23S rRNA, twelve different mutations were found in *gyrA*, ten mutations were found in *cmeR*, and one mutation was found in *rpsL*. The most prevalent in the 23S rRNA gene was a nucleotide change at position 1752 from T to C that was not predicted to result in an amino acid substitution (n = 28), in *gyrA* it was an amino acid change at position 285 from R to K (n = 23), in *cmeR* it was an amino acid change at position 144 from G to D (n = 16), while in *rpsL* it was an amino acid change at position 126 from A to T (n = 11). Additionally, nine isolates carried a mutation in *gyrA* that results in a stop codon at position 863; however this was not manifested in ciprofloxacin resistance phenotypically.

### 3.6 Insertion sequences (IS)

A total of eight insertion sequences were detected in the genomes analysed in this study (**Table 3**). Among them, four belonged to *C*. *coli*, and the remaining four belonged to *Helicobacter pylori*, *Methanosaeta thermophila*, *Staphylococcus aureus*, and *Treponema denticula*. The most commonly recovered IS was ISCco1, from *C*. *coli*, followed by IS606 from *H*. *pylori*. Interestingly, IS606 was more prevalent in clinical isolates (n = 7) than broiler isolates (n = 2).

**Table 3:** Prevalence of insertion sequences (IS) detected in clinical and broiler genomes in this study.

IS Origin	IS ID	Source		Total (n = 30)
		Clinical (n = 15)	Broiler (n = 15)	
*C*. *coli*	ISCac1	0 (0.00%)	1 (6.67%)	1 (3.33%)
	ISCco1	9 (60.00%)	8 (53.33%)	17 (56.67%)
	ISCco2	2 (13.33%)	1 (6.67%)	3 (10.00%)
	ISCco4	0 (0.00%)	2 (13.33%)	2 (6.67%)
*Helicobacter pylori*	IS606	7 (46.67%)	2 (13.33%)	9 (30.00%)
*Methanosaeta thermophila*	ISMth1	1 (6.67%)	0 (0.00%)	1 (3.33%)
*Staphylococcus aureus*	ISSau6	1 (6.67%)	1 (6.67%)	2 (6.67%)
*Treponema denticola*	ISTde1	3 (20.00%)	2 (13.33%)	5 (16.67%)

## 4. Discussion

In this study, our objective was to characterise and compare a small subset of the *C*. *jejuni* strains that have circulated in the Irish poultry sector and that have caused illness in the human population. Genetically similar clinical and broiler *C*. *jejuni* isolates were identified. Indeed, the most common clonal complex, ST-45, was identified across clinical and broiler isolates, suggesting that it may be transmitted from broilers to humans. Strains that share a clonal complex have originated from a common ancestor, and the resulting genetic relatedness often leads to shared phenotypic properties including niche association and virulence [39]. Notably, the ST-45 clonal complex is highly genetically diverse, and appears in a wide variety of hosts and geographical locations. It is the second most common clonal complex in the PubMLST database for *C*. *jejuni*, with a prevalence of 9.2% [25]. The prevalence of ST-45 in Ireland was previously estimated to be approximately 4.9% in clinical and 8.5% in broiler isolates [40, 41], while in this study it accounted for 16.7% of both clinical and broiler isolates. Additionally, this clonal complex was previously found to be prevalent in post-infectious inflammatory bowel disease [42].

Other clonal complexes associated with broilers (ST-257, ST-354, and ST-661) [43–45], cattle (ST-61 and ST-42) [46], and another host generalist (ST-21) were also identified [47]. Interestingly, while ST-21 is found in a wide variety of niches, in this study it was only found in clinical isolates, most likely because of our limited sample size (n = 15). Previous reports found that ST-21 was the most common clonal complex in clinical and broiler *C*. *jejuni* isolates in Ireland, accounting for approximately 22% and 17.1% isolates, respectively, which is consistent with the PubMLST database entries, whereby the clonal complex ST-21 accounts for the majority (23.3%) of all entries [25, 40, 41]. However, in this study ST-45 was more prevalent. Furthermore, complexes ST-22 and ST-257, which were found in broilers in this study, are reportedly associated with severe illness in humans [42, 48].

The detection of two cattle-associated lineages (ST-61 and ST-42) in broiler isolates indicates possible transmission from cattle to broilers [46, 49]. Previous studies have found that broiler houses near livestock are at greater risk of contamination with *Campylobacter* [50]. Two clinical isolates were also classified as ST-61, which were genetically similar to the broiler ST-61 isolate, suggesting that although ST-61 is more commonly associated with cattle, it may also be detected in and transmitted to broilers and humans. ST-61 has been previously detected in broilers, which supports this observation [49]. Nevertheless, given the previously reported higher prevalence of ST-61 in cattle it remains a more likely source of transmission of this clonal complex to humans [51].

The genomes in this study revealed an open pangenome (Fig 2), whereby with the addition of each new genome, new genes were identified. An open pan-genome is associated with a sympatric lifestyle, along with large genomes and a high rate of horizontal gene transfer [52]. In this study, the accessory genome of *C*. *jejuni* accounted for 59.4% of its pan-genome. A large accessory genome is associated with niche versatility, while a small accessory genome is associated with niche specificity [52]. *C*. *jejuni* is known for its ability to colonise a wide variety of hosts and niches [53], which would require adaptation to a wide range of environments. Niche versatility can be advantageous in agricultural settings, where the close proximity of multiple mammalian and avian animals may provide multiple opportunities for zoonotic transmission [54].

The most prevalent functions in the core genome of *C*. *jejuni* were translation, amino acid metabolism and energy production. *C*. *jejuni* shows preference towards consuming amino acids including glutamate, aspartate, serine and proline [55]. Hence, the conservation of genes associated with amino acid metabolism is consistent with the specificity that *C*. *jejuni* phenotypically displays in its metabolism of amino acids [55]. Indeed, as it cannot metabolise glucose, amino acids are an important energy source [55]. Similarly, energy production relies on conserved pathways such as the citric acid cycle [56]. Translation is also a conserved biological process, and it has been previously reported to be enriched in the essential genome of *C*. *jejuni* [57]. Furthermore, these categories were also among the most prevalent functions in the core genome of *C*. *jejuni* in a previous report, along with cell wall biogenesis, post-translation modification, and carbohydrate and inorganic ion metabolism [58].

The accessory genome was significantly associated with functions including cell wall biogenesis, DNA replication, recombination and repair, carbohydrate metabolism, and intracellular trafficking. *C*. *jejuni* has a heterogenous cell wall composition, comprised of diverse LOS classes, some of which are associated with severe illness [59]. A diverse accessory genome comprising carbohydrate transport and metabolism and cell wall biogenesis would facilitate this, as many genes involved in glycosylation would fall into these categories. Additionally, variation in the genes associated with DNA replication, recombination and repair could facilitate genomic adaptation in different environments [9]. Previous research has also found that cell wall biogenesis and carbohydrate metabolism were amongst the most represented categories in the accessory genome of *C*. *jejuni* [58]. However, in that study fewer genes were associated with DNA replication, recombination and repair and intracellular trafficking in comparison to our results. Such differences are not unprecedented, as the accessory genome is highly variable. Indeed, as a result of this diversity, the majority of the accessory genome of *C*. *jejuni* is uncharacterised, unlike the core genome, which is mostly comprised of genes of known function (**Table 2**).

While no significant differences were identified between the core and accessory genomes of clinical and broiler isolates, in this study the unique genome of broiler *C*. *jejuni* isolates was associated with significantly more genes involved in carbohydrate and lipid metabolism, secondary metabolite synthesis, translation and cell wall biogenesis than the unique genome of clinical isolates. Notably, at this level of the analysis the results can be prone to noise and further confirmation with larger *C*. *jejuni* populations would be advantageous. While to our current knowledge no such observations have been previously reported, it has been shown that the genome size of *C*. *hepaticus*, which causes spotty liver disease in chickens, is smaller than the *C*. *jejuni* and *C*. *coli* genomes, which may be attributed to niche specialisation [60]. In contrast, no difference in genome size was reported for the generalist *C*. *jejuni* clonal complex ST-21 compared to the cattle lineage ST-61 between 1979 and 2013 [46]; however multiple recombination events have been described. It is broadly accepted that host specialisation is associated with gene loss due to a tendency towards the deletion of redundant or superfluous genes, which could indicate that some of the broiler isolates in this study have the capacity to colonise different niches [61, 62].

The distribution of virulence genes across clinical and broiler *C*. *jejuni* strains in this study was not statistically different suggesting that all *C*. *jejuni* strains in this study had the potential to cause human illness. Notably however, genes associated with severe illness including *neuABC*, *wlaN* and *cstIII* were only detected in clinical isolates. These genes encode sialyltransferases, which form structures on the surface of the *C*. *jejuni* LOS that resemble gangliosides, inducing the production of cross-reactive anti-ganglioside antibodies that lead to the development of autoimmune neuropathies such as Guillain Barré Syndrome [63]. In total, eight virulence genes associated with LOS synthesis were present in clinical *C*. *jejuni* strains but were absent from the broiler strains, suggesting that LOS variation may be an important adaptation mechanism for *C*. *jejuni* colonisation of human hosts. Furthermore, while *neuABC*, *wlaN* and *cstIII* were not identified in broiler *C*. *jejuni* in this study, they have been previously found in poultry isolates, albeit less often than in clinical isolates [64]. An ABRicate search equipped with VFDB for these genes in the publicly available dataset of 122 clinical Irish *Campylobacter spp*. isolates by Redondo et al (2019) revealed a prevalence of 18%, 18.8%, 17.2%, 13%, and 7.4% for *neuA1*, *neuB1*, *neuC1*, *cstIII* and *wlaN*, respectively, which is close to the prevalence observed in this study (*wlaN* = 13.3%, *cstIII*, *neuABC* = 20%). Additionally, a *H*. *pylori* transposon (IS606) was identified in clinical isolates more frequently than in broiler isolates (**Table 3**). This transposon was previously identified in *Campylobacter spp*. in association with aminoglycoside resistance [65, 66]. In this study, its prevalence in clinical isolates could indicate possible public health significance.

Interestingly, only three virulence genes from broilers were absent from clinical *C*. *jejuni*. Two of these genes encode amino acid ABC transporter permease protein (PEB1), which has been previously associated with increased invasion and colonisation of chicks [67]. The third encodes a galactosyltransferase component of LOS synthesis, *galT*, and reportedly may affect biofilm formation [68]. While these genes may confer an advantage for chick colonisation, they may not be essential for human colonisation. Indeed, we found that they had a low prevalence of 9.8% (*CJE0998*), 12.5% (*JJD26997_0895*), and 4.5% (*CJJ81176_1165*) in the clinical isolates from Redondo et al (2019), which reflects that they are likely not essential for human infection, however they were present in these isolates, which indicates that they are not exclusively found in broiler isolates.

We observed a discrepancy between WGS and conventional PCR (cPCR) results in this study that may be due to differences in resolution and/or sensitivity between the two assays, and/or the presence of polymorphisms on the target genes. Higher sensitivity in real time PCR has been previously reported in comparison to cPCR [69–71], and it is possible that a similar effect may result in the difference in product detection between WGS and cPCR. Additionally, primer-based amplification of virulence genes that are prone to variation could lead to a lower recovery rate due to polymorphisms at targets sites for amplification [72]. Nevertheless, cPCR is a quick, accessible and cost effective tool that reliably recovers genes that are genetically stable. Indeed, the recovery rate of *tet(O)* via cPCR compared to WGS was slightly higher. The presence of a conventional PCR positive *tet(O)* strain (CJB26) that was tetracycline susceptible and *tet(O)* negative via WGS screening suggests that the isolate may have lost this gene during cultivation, or it may have been a false positive. Additionally, the absence of this gene from CJB26 was further confirmed via *in silico* PCR using the same primers [73]. Loss of *tet(O)* has been previously reported in *Enterococcus spp*. after *in vitro* digestion [74], and segregational loss of plasmid-encoded genes during replication could also lead to the loss of plasmid-encoded *tet(O)* [75, 76]. Nevertheless, it would be advantageous to further investigate the differences between cPCR and WGS as the former is a routinely used screening tool, and WGS is frequently used for in-depth analyses and comparisons.

In this study, antimicrobial resistance determinants associated with β-lactam, tetracycline, fluoroquinolone, and aminoglycoside resistance were identified, as well as the presence of the *cmeABC* operon, *cmeR*, and various point mutations in 23S rRNA, *gyrA*, *cmeR*, and *rpsL* genes. The presence of tetracycline and ciprofloxacin resistance determinants matched the previously established phenotypic resistance profiles exactly. Additionally, multidrug resistance was found in two clinical and one broiler isolate, with genotypic resistance determinants for β-lactams, tetracycline, ciprofloxacin and streptomycin (n = 1) and for β-lactams, tetracycline, and quinolones (n = 2). Furthermore, it is possible that the previously unreported stop codon detected in *gyrA* (Q863*) did not affect cell growth because it prematurely terminates *gyrA* by a single amino acid at the end of the amino acid sequence. Additionally, based on the phenotypic susceptibility of the affected isolates it is likely not enough to confer fluoroquinolone resistance. To our knowledge, this *gyrA* mutation has not been previously reported. All determinants and drug resistance profiles detected in this study were recently detected in Irish isolates [77], with the exception of *bla*_*OXA-184*_, *bla*_*OXA-447*_ and *bla*_*OXA-449*_, although they have been previously reported in Italy [78].

## 5. Conclusion

In this study, strains belonging to the ST-45 clonal complex were the most prevalent and included a combination of clinical and broiler strains, suggesting possible transmission between the two niches. Other genetically similar strains could also be at risk for transmission between broilers and humans. The prevalence of lineages associated with severe human illness may pose a risk to public health. The presence of a cattle-associated lineage (ST-61) in broiler and clinical samples reaffirms that *C*. *jejuni* from adjacent livestock may be a contamination risk for broiler farms, which can also be transmitted to humans.

Genes associated with amino acid metabolism, energy production, and translation were abundant in the core genome of *C*. *jejuni*, which may be indispensable for *C*. *jejuni* survival, while genes associated with cell wall biogenesis, carbohydrate metabolism, DNA recombination, and intracellular trafficking were associated with the accessory genome, and may be important for *C*. *jejuni* niche adaptation. While the core and accessory genomes of broiler and clinical isolates were not significantly different, those from broilers carried a higher number of genes that were absent from clinical isolates, which could be important for survival and persistence in broiler environments.

No significant difference in the virulence or antimicrobial resistance of broiler and clinical isolates was detected; however genes associated with severe illness (*neuABC*, *wlaN*, and *cstIII*) were only found in clinical isolates in this study, which may be the result of the small sample size or may indicate that the incidence of hyper-virulent *C*. *jejuni* in poultry remains low, but further research is required to firmly establish if this observation holds true for all broiler and clinical isolates.

## Supporting information

S1 Appendix Distance matrices of clinical and broiler genomes. (DOCX)

S2 Appendix Functional annotation of all, clinical and broiler genomes. (ZIP)

S3 Appendix Virulence and antimicrobial resistance determinant screening outputs. (ZIP)
