# Supplementary material for: Genetic characterisation of a subset of Campylobacter jejuni isolates from clinical and poultry sources in Ireland
Source: PLoS One. 2021 Mar 9;16(3):e0246843. doi: 10.1371/journal.pone.0246843 (PMC7943001; doi:10.1371/journal.pone.0246843)
Supplement: S1 Appendix — (DOCX) [file pone.0246843.s001.docx]

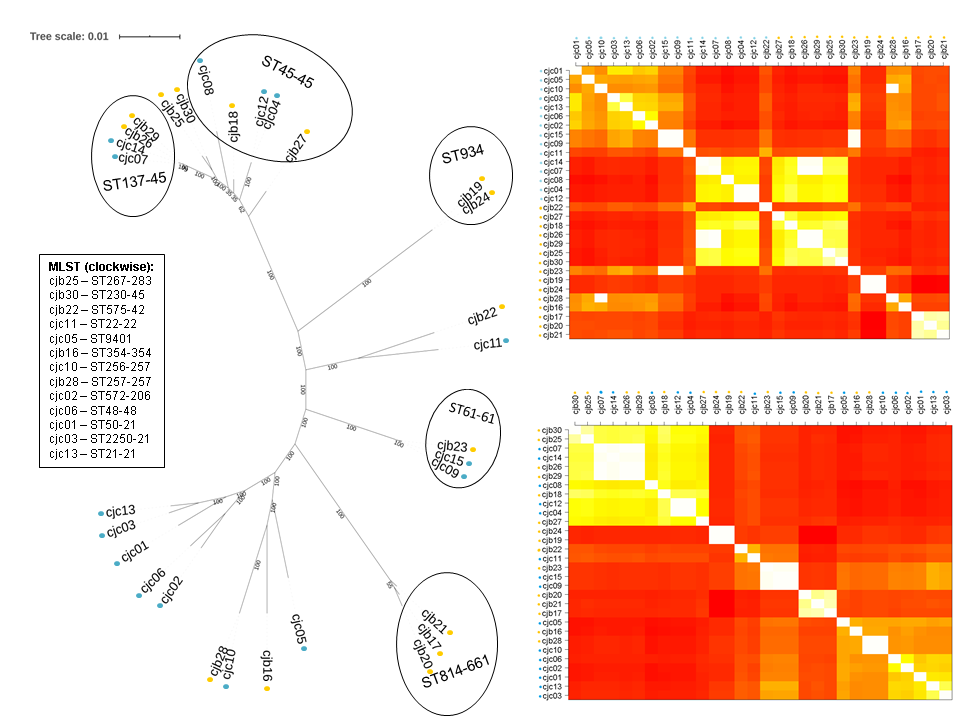


**B.**

**A.**

**Fig. 1**: Distance matrices of clinical and broiler isolates arranged by source (A), and aligned to the maximum likelihood tree (B). Distance is arranged from red (least similar) to white (most similar). Isolates CJC01 - CJC15 were recovered from clinical human infection (blue dots), and CJB16 - CJB30 were recovered from broilers (orange dots).
