# Supplementary material for: Genetic characterisation of a subset of Campylobacter jejuni isolates from clinical and poultry sources in Ireland
Source: PLoS One. 2021 Mar 9;16(3):e0246843. doi: 10.1371/journal.pone.0246843 (PMC7943001; doi:10.1371/journal.pone.0246843)
Supplement: S2 Appendix — (ZIP) [file pone.0246843.s002.zip › S2 Appendix/Extended Table 2.docx]

**Table 2:** Functional annotation of the pan, core and accessory genomes and of genes unique to clinical and broiler *C. jejuni* isolates. Percentages represent the prevalence of genes within their respective pan, core, accessory or unique genome category.

| **COG Category** | **Function** | **All** | | | **Broiler** | | | | **Clinical** | | | |
| --- | --- | --- | --- | --- | --- | --- | --- | --- | --- | --- | --- | --- |
|  |  | **Pan (n = 2789)** | **Core (n = 1301)** | **Accessory (n = 1487)** | **Pan (n = 2789)** | **Core (n = 1319)** | **Accessory (n = 1289)** | **Unique (n = 419)** | **Pan (n = 2410)** | **Core (n = 1340)** | **Accessory (n = 1070)** | **Unique (n = 223)** |
| A | RNA processing and modification | 1(0.0%) | 1(0.1%) | 0(0.0%) | 1(0.0%) | 1(0.1%) | 0(0.0%) | 0(0.0%) | 1(0.0%) | 1(0.1%) | 0(0.0%) | 0(0.0%) |
| C | Energy production and modificaiton | 148(5.3%) | 116(8.9%)***^, a^ | 32(2.2%) | 145(5.2%) | 117(8.9%)***^, a^ | 28(2.2%) | 7(1.7%) | 142(5.9%) | 118(8.8%)***^, a^ | 24(2.2%) | 5(2.2%) |
| D | Cell cycle control | 29(1.0%) | 17(1.3%) | 12(0.8%) | 25(0.9%) | 17(1.3%)***^, a^ | 8(0.6%) | 3(0.7%) | 27(1.1%) | 17(1.3%)*^, a^ | 10(0.9%) | 4(1.8%) |
| E | Amino acid transport and metabolism | 178(6.4%) | 118(9.1%)***^, a^ | 60(4.0%) | 177(6.3%) | 118(8.9%)***^, a^ | 59(4.6%) | 8(1.9%) | 175(7.3%) | 118(8.8%)***^, a^ | 57(5.3%) | 6(2.7%) |
| F | Nucleotide transport and metabolism | 80(2.9%) | 69(5.3%)***^, a^ | 11(0.7%) | 78(2.8%) | 69(5.2%)***^, a^ | 9(0.7%) | 2(0.5%) | 79(3.3%) | 69(5.1%)***^, a^ | 10(0.9%) | 2(0.9%) |
| G | Carbohydrate transport and metabolism | 101(3.6%) | 38(2.9%) | 63(4.2%)*^, a^ | 98(3.5%) | 38(2.9%) | 60(4.7%)***^, a^ | 17(4.1%)*^, e^ | 89(3.7%) | 38(2.8%)***^, c^ | 51(4.8%)***^, b^ | 6(2.7%) |
| H | Coenzyme transport and metabolism | 121(4.3%) | 80(6.1%)***^, a^ | 41(2.8%) | 114(4.1%) | 80(6.1%)***^, a^ | 34(2.6%) | 13(3.1%) | 111(4.6%) | 83(6.2%)***^, a^ | 28(2.6%) | 8(3.6%) |
| I | Lipid transport and metabolism | 60(2.2%) | 29(2.2%) | 31(2.1%) | 55(2.0%) | 29(2.2%)***^, b^ | 26(2.0%)***^, c^ | 11(2.6%) | 49(2.0%) | 30(2.2%)***^, b^ | 19(1.8%)***^, c^ | 5(2.2%) |
| J | Translation, ribosomal structure and biogenesis | 188(6.7%) | 138(10.6%)***^, a^ | 50(3.4%) | 184(6.6%) | 139(10.5%)***^, a^ | 45(3.5%) | 14(3.3%)*^, e^ | 174(7.2%) | 140(10.4%)***^, a^ | 34(3.2%) | 5(2.2%) |
| K | Transcription | 77(2.8%) | 40(3.1%) | 37(2.5%) | 72(2.6%) | 41(3.1%)***^, b^ | 31(2.4%)***^, c^ | 9(2.1%) | 71(2.9%) | 43(3.2%)***^, b^ | 28(2.6%)***^, c^ | 5(2.2%) |
| L | DNA replication, recombination and repair | 153(5.5%) | 60(4.6%) | 93(6.3%)***^, a^ | 137(4.9%) | 61(4.6%)***^, c^ | 76(5.9%)***^, b^ | 29(6.9%)*^, e^ | 128(5.3%) | 64(4.8%)***^, d^ | 64(6.0%)***^, d^ | 15(6.7%) |
| M | Cell wall/membrane biogenesis | 297(10.6%) | 94(7.2%) | 203(13.7%)***^, a^ | 273(9.8%) | 96(7.3%) | 177(13.7%)***^, a^ | 53(12.6%)**^, e^ | 248(10.3%) | 94(7.0%)***^, c^ | 154(14.4%)***^, b^ | 26(11.7%) |
| N | Cell motility | 91(3.3%) | 55(4.2%)*^, a^ | 36(2.4%) | 89(3.2%) | 53(4.0%)***^, b^ | 36(2.8%)***^, c^ | 9(2.1%) | 87(3.6%) | 54(4.0%)***^, b^ | 33(3.1%)***^, c^ | 5(2.2%) |
| O | Post-translational modification, protein turnover, and chaperones | 76(2.7%) | 60(4.6%)***^, a^ | 16(1.1%) | 73(2.6%) | 61(4.6%)***^, a^ | 12(0.9%) | 1(0.2%) | 75(3.1%) | 61(4.6%)***^, a^ | 14(1.3%) | 3(1.3%) |
| P | Inorganic ion transport | 164(5.9%) | 92(7.1%)*^, a^ | 67(4.5%) | 159(5.7%) | 96(7.3%)***^, b^ | 59(4.6%)***^, c^ | 15(3.6%) | 150(6.2%) | 99(7.4%)***^, a^ | 44(4.1%) | 9(4.0%) |
| Q | Secondary metabolite biosynthesis, transport and catabolism | 55(2.0%) | 12(0.9%) | 43(2.9%)***^, a^ | 54(1.9%) | 13(1.0%) | 41(3.2%)***^, a^ | 14(3.3%)**^, e^ | 43(1.8%) | 13(1.0%) | 30(2.8%)***^, a^ | 3(1.3%) |
| S | Function unknown | 585(21.0%) | 184(14.1%) | 401(27.0%)***^, a^ | 523(18.8%) | 191(14.5%) | 332(25.8%)***^, a^ | 107(25.5%)**^, e^ | 485(20.1%) | 192(14.3%) | 293(27.4%)***^, a^ | 70(31.4%) |
| T | Signal transduction | 59(2.1%) | 40(3.1%)***^, a^ | 8(0.5%) | 56(2.0%) | 40(3.0%)***^, c^ | 6(0.5%)***^, b^ | 5(1.2%) | 57(2.4%) | 40(3.0%)***^, a^ | 7(0.7%) | 2(0.9%) |
| U | Intracellular trafficking, secretion and vesicular transport | 96(3.4%) | 42(3.2%) | 67(4.5%)*^, a^ | 85(3.0%) | 42(3.2%)***^, c^ | 54(4.2%)***^, b^ | 18(4.3%) | 82(3.4%) | 43(3.2%)***^, c^ | 50(4.7%)***^, b^ | 16(7.2%) |
| V | Defence mechanisms | 60(2.2%) | 12(0.9%) | 48(3.2%)***^, a^ | 58(2.1%) | 12(0.9%) | 46(3.6%)***^, a^ | 16(3.8%) | 44(1.8%) | 12(0.9%) | 32(3.0%)***^, a^ | 7(3.1%) |
| N/A | Uncharacterised | 337(12.1%) | 94(7.2%) | 243(16.3%) | 337(12.1%) | 94(7.1%) | 215(16.7%) | 94(22.4%) | 242(10.0%) | 97(7.2%) | 145(13.6%) | 32(14.3%) |

* = p < 0.05; * = p < 0.01; *** = p < 0.001; a = chi square test was only significantly positively associated with this value; b and c = both values were significantly positively associated, where b showed a stronger association than c; d = both values were significantly positively associated equally, more so than the third value; e = significant differences in comparisons between clinical and broiler genomes.
