# Supplementary material for: Genetic characterisation of a subset of Campylobacter jejuni isolates from clinical and poultry sources in Ireland
Source: PLoS One. 2021 Mar 9;16(3):e0246843. doi: 10.1371/journal.pone.0246843 (PMC7943001; doi:10.1371/journal.pone.0246843)
Supplement: S2 Appendix — (ZIP) [file pone.0246843.s002.zip › S2 Appendix/Fig.1accumulationcurve.docx]

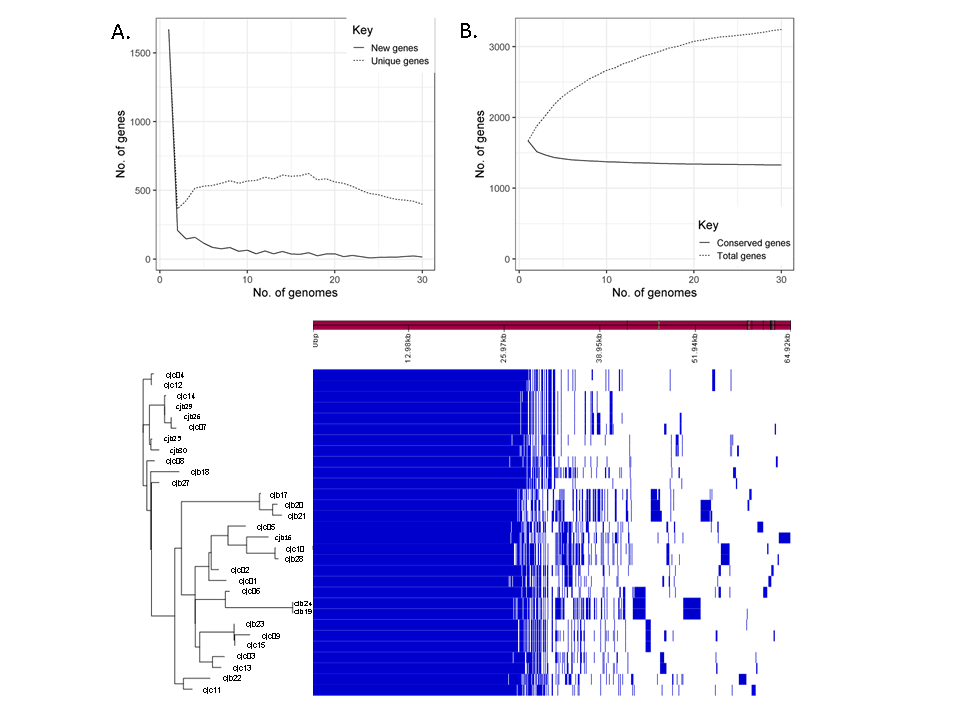


**Fig. 1**: Accumulation curve of conserved and total genes with each genome that is added to the dataset (A), accumulation curve of new and unique genes with each genome that is added to the dataset (B).
